# The complete mitochondrial genome of Dapulian pig (*Sus scrofa*) and a phylogenetic study

**DOI:** 10.1080/23802359.2018.1443036

**Published:** 2018-02-23

**Authors:** Hongyu Lu, Gen Liu, Yanchao Wang, Yunliang Jiang

**Affiliations:** a Department of Genetics, College of Agronomy, Shandong Agricultural University, Taian, China;; b Shandong Provincial Key Laboratory of Animal Biotechnology and Disease Control and Prevention, College of Animal Science and Veterinary Medicine, Shandong Agricultural University, Taian, China

**Keywords:** Dapulian pig, mitochondrial, genome, phylogeny

## Abstract

The two types of mitochondrial genomes of Dapulian pig has been sequenced and annotated completely. The entire genome is 16,610 bp in length with an A + T content of 60.6% (GenBank accession No. MG009445, MG009446). This mitochondrial genome consisted of 13 protein-coding genes, 2 ribosomal RNA subunits and 22 transfer RNAs, and 1 D-loop control regions. A phylogenetic tree with the near-complete mitochondrial genome sequences of two types of Dapulian pigs together with 17 other Chinese pig breeds was constructed. The results can be subsequently used to provide information for pig phylogenetic and insights into the evolution of genomes. The mitochondrial genome would play an important role in population genetics and phylogenetic analysis of *Sus scrofa*.

As one of the relatively large black pigs of North China, the Dapulian pigs are mainly distributed in Jining and Heze districts of Shandong Province, characterized by excellent germplasm characteristics such as resistance to both disease and rough feed, and listed as the protected breeds of the national livestock and poultry genetic resources in China. Studies indicated that Dapulian pigs were more resistance to porcine reproductive and respiratory syndrome (PRRS) virus infection than commercial Duroc Landrace Yorkshire (DLY) hybrid swines (Jiang et al. 2012; Li et al. 2014; Hu et al. 2016). The complete mitochondrial genome sequence of Dapulian pig has not yet been reported. In this study, we present the complete mitochondrial genome of Dapulian pig (GenBank accession no: MG009445, MG009446, NCBI Reference Sequence: NC_000845.1).

The specimen was collected from the Dapulian pig preservation centre (Jining, Shandong, China). The specimen (DNA and tissue) was deposited in the Shandong Provincial Key Laboratory of Animal Biotechnology and Disease Control and Prevention (Voucher numbers DPL-20150043; DPL-20150083). Total genomic DNA of ear tissue was extracted from the ear tissue using a TIANamp Genomic DNA Kit (Tiangen Biotech, Beijing, China) according to the manufacturer’s protocol. The concentration and purity of genomic DNA were determined by absorbance at 260 and 280 nm and the ratio of 260/280 with a UV spectrophotometer (Eppendorf, Hamburg, Germany). Whole genomic sequencing was performed by Boshang Biotechnology Co., Ltd (Shanghai, China) using ABI3730 DNA sequencer.

The complete sequence of mitochondrial DNA of Dapulian pig is 16,610 bp in length. The overall nucleotide composition is 34.8% A, 25.8% T, 26.2% C, and 13.2% G, with a total A + T content of 60.6%, that is heavily biased toward A and T nucleotides. It presents the typical set of 37 genes, including 13 PCGs (*cox1-3*, *cytb*, *nad1-6*, *nad4L*, *atp6*, and *atp8*), 22 tRNA genes, 2 genes for ribosomal RNA subunits (12SrRNA,16SrRNA), and a major non-coding control region (D-Loop region). The gene arrangement in the mitochondrial genome of Dapulian pig is conserved as other pigs in Mammalia. In the mt genome of Dapulian pig, furthermore, the whole mitogenome sequence has six overlaps, a total of 72 bp overlaps have been found at six gene junctions of the genome (*atp8* and *atp6* share 43 nucleotides; *atp6* and *cox3* share 1 nucleotide; *nad4* and *nad4L* share 7 nucleotides; *nad5* and *nad6* share 17 nucleotides; *tRNA-Ile* and *tRNA-Gln* share 3 nucleotides; *tRNA-Cys* and *tRNA-Tyr* share 1 nucleotide).

The 19 same length (15977 bp,including 13 PCGs, 21 tRNA genes, 2 genes for ribosomal RNA subunits, and partial D-Loop region) sequences of mtDNA obtained from type I and type II Dapulian pigs and 17 other Chinese pig breeds were used to construct phylogenetic tree by using MEGA5.0 with 1000 bootstrap replicates. In this study, through the construction of the NJ phylogenetic tree based on the near-whole mitochondrial genome (Figure 1), we found that the Dapulian pig may have two maternal origins. Type I Dapulian and Laiwu black pig have a close genetic relationship; this result was consistent with the geographical distribution. A more close genetic relationship was observed between the Type II Dapulian pig and the Wannanhua pig (belong to Anhui Province).

**Figure 1.**
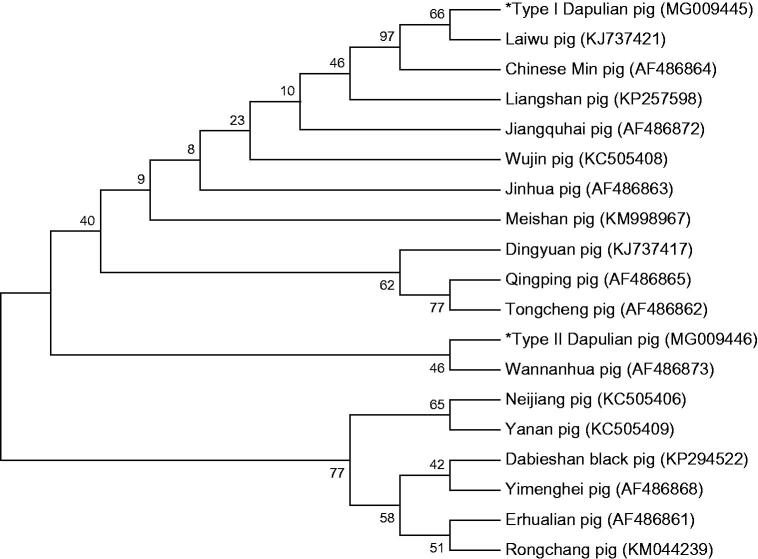
Phylogenetic relationships of Dapulian pig and 17 other pig breeds based on the near-whole mitochondrial genome using NJ method.
